# Effects of different slaughter weights on meat quality parameters and gene expression related to fat metabolism in muscles, liver, and gluteal fat in Lalahan lambs

**DOI:** 10.5194/aab-69-397-2026

**Published:** 2026-08-07

**Authors:** Rabia Arslan, Necmettin Ünal, Akın Yakan

**Affiliations:** 1 Ankara University, Institute Health Science, Department of Animal Breeding and Husbandry, 06110, Ankara, Türkiye; 2 Ankara University, Faculty of Veterinary Medicine, Department of Animal Breeding and Husbandry, 06110, Ankara, Türkiye

## Abstract

The study examined the meat quality characteristics and expression levels of 3 genes (*SREBP-1c, PPAR* γ , and *FASN*) affecting fatty acid composition in Lalahan lambs at different slaughter weights. Thirty-six male lambs were randomly divided into three groups and fed intensively. Six lambs from each group were slaughtered at average slaughter weights of 35 (Gr35), 40 (Gr40), and 45 kg (Gr45), respectively. Slaughter weight had a significant effect ( P<0.05 ) on pH and $a^{*}$ index from color parameters at 24 h post-mortem, but there was no significant effect on ΣPUFA/ΣSFA , ΣUFA/ΣSFA , ω6/ω3 , AI, and TI values. There were no significant differences between groups in terms of the expression of *SREBP-1c* and *PPAR* γ genes in either liver or Musculus longissimus dorsi lumbalis (MLD) tissues, whereas the difference in expression was significant for the *FASN* gene in liver and MLD ( P<0.05 ). There were no significant differences between groups in terms of the expression of the three genes in gluteal adipose tissue. The findings indicate that Lalahan lambs exhibit favorable meat quality traits and lipid metabolism gene expression patterns under intensive feeding conditions. Comparisons with previously published studies suggest that these characteristics may be somewhat better than those reported for Kivircik sheep (Lalahan sheep are a breed derived from Kivircik sheep); however, studies conducted under the same experimental conditions are needed to make direct comparisons among breeds. In short, high-quality meat can be produced from lambs of the Lalahan sheep genotype.

## 1 Introduction

In sheep breeding, sheep meat production is the most important source of income. In order to maximize sheep meat production, it is necessary to obtain a high parturition rate with high viability and quickly bring lambs to slaughter weight. To produce high-quality sheep meat, it is necessary to have a high enough milk yield for the lambs, while, in sheep, it is essential to have good nutrition, carcass quality characteristics, and meat quality characteristics with high viability (Unal et al., 2006).

Lalahan sheep, which were obtained by combination melting between the Kivircik and Akkaraman breeds, contain approximately 75 % Kivircik genotype and 25 % Akkaraman genotype (Akçapınar et al., 2004; Erol, 2013). Several studies have investigated Lalahan sheep product characteristics in the first years of breeding, single lactation, and single slaughtering weight. However, no studies have investigated milk yield, milk quality, breast types, and breast sizes in sheep with different lactations, nor have they investigated yield performance, cutting, carcass, and meat quality characteristics or the levels of expression of genes affecting the quality of meat in different weight cuts. While Erol et al. (2017) studied the genotype of parturition rate and several morphological characteristics, no study has examined meat quality and the levels of expression of genes potentially affecting these parameters in sheep at different slaughtering weights.

Meat quality is influenced by several factors, such as breed, gender, age, nutrition, slaughtering weight, and pre-slaughtering stress. Meat quality is evaluated in terms of various criteria, including pH, color, water-holding capacity, cooking loss, and fatty acid composition (Yakan and Ünal, 2010).

The relationships between genes and yield characteristics in farm animals have been intensively investigated, with one area being the levels of expression of genes that affect meat yield and quality. in particular, many genes related to lipid metabolism have been identified. These include genes involved in controlling the energy that lambs take from their feed, which can affect the composition of fatty acids in the body, and genes involved in regulating lipid metabolism, such as *SREBP-1c* (sterol regulator element binding protein 1c), *PPAR* (peroxisome proliferator-activated receptor gamma), and *FASN* (fatty acid synthase) (Deng et al., 2016; Fernández-Alvarez et al., 2011; Yakan et al., 2021). Accordingly, the present study examined the meat quality characteristics in Lalahan lambs and the levels of expression of three genes (*SREBP-1c, PPAR* γ , and *FASN*) known to affect the composition of fatty acids at three different slaughter weights (35, 40, and 45 kg ).

## 2 Material and method

### 2.1 Animal material

The animal material was male Lalahan sheep genotype lambs raised in the Sheep and Goat Breeding Unit of the Lalahan International Animal Research and Training Center Directorate. Thirty-six male sheep, weaned at 90 d and with starting weights of approximately 20 kg , were randomly divided into three groups. The lambs were then fed intensively according to the schedule in Table 1 until they reached approximately the target living weight for each group, namely 35 (Gr35), 40 (Gr40), and 45 kg (Gr45). From each group of 12, 6 lambs were selected for slaughter (total of 18 lambs divided into three groups). Although the animals selected for slaughter represented the average live weight of the groups, they were considered to be a limitation of the study. Feeding was stopped for 12 h before slaughtering, but access to water was allowed until slaughtering. Each animal was weighed immediately before slaughtering.

**Table 1:** Nutrient content of concentrate feed and alfalfa hay.

Composition	Concentrate	Alfalfa
	feed	hay
Dry matter (%)	90.87	91.96
Crude protein (%)	16.42	13.72
Crude cellulose (%)	11.81	38.74 (ADF)
Crude oil (%)	1.68	1.76
Crude ash (%)	6.78	12.71
Metabolic energy ( $\mathrm{kcal}\;{\mathrm{kg}}^{-1}$ )	2700	2080
Fatty acid value	Concentrate feed	Alfalfa hay
Σ SFA	44.17	47.38
Σ MUFA	51.94	49.41
Σ PUFA	3.89	3.21
Σ UFA	55.83	52.62
ΣPUFA/ΣSFA	0.088	0.067
ΣUFA/ΣSFA	1.264	1.110
Σω6	3.322	2.207
Σω3	0.5676	1.002
ω6/ω3	5.852	2.201
Nutrition value (NV)	3.304	2.299
Atherogenic index (AI)	0.472	0.548
Thrombogenic index (TI)	0.865	0.899
Ingredients	%	
Barley	62	
Bran	17.4	
Sunflower meal	18	
Limestone	2	
Salt	0.5	
Vit-Min premix	0.1	
(Rovimix 302 FM/30)		

NV =(C18:0+C18:1)/C16:0 .AI =(C12:0+(4⋅C14:0)+C18:0)/ΣUFA .TI =(C14:0+C16:0+C18:0)/(0.5⋅C18:1)+(0.5⋅ΣMUFA)+(0.5⋅Σω6)+(3⋅Σω3)+(Σω3/Σω6) .

### 2.2 Meat quality analyses

To determine meat quality, the pH, color, water retention capacity, cooking loss, and fatty acid composition characteristics were measured, as detailed below.

The pH was measured with a portable pH meter (Mettler Toledo SG2) from Musculus longissimus dorsi lumbalis (MLD) and Musculus semimembranosus (MSM) samples using a glass electrode (Inlab 427). Measurements were taken from meat samples stored at +4 °C at 15 and 60 min and 24 h after slaughtering.

Meat color was measured using a colorimeter (Konika Minolta CR-400 Chromometer) using the $L^{*}$ , $a^{*}$ , and $b^{*}$ systems (Honikel, 1998). Measurements were taken from the fatless parts of the cut surfaces of MLD and MSM samples at 15 and 60 min and 24 h after slaughter. After the carcasses were aged for 24 h , prior to instrumental color measurements, the freshly cut meat surfaces were allowed to undergo the “bloom” process for 30 min by exposing them to atmospheric oxygen at 4 °C .

Water-holding capacity was measured at 24, 48, and 72 h after cutting. A 5 g MLD meat sample was divided into five pieces, and each piece was weighed using an electronic scale, sensitive to 0.001 g . Each piece was then placed between two layers of glass and filter paper, and a weight of 2250 g was placed on top for 5 min . The meat pieces were then removed and reweighed. The sample's percentage water-holding capacity was calculated as the difference between the weight before and after compression (Barton-Gade et al., 1994).

Cooking loss was measured 24 and 48 h after slaughter. A 50 g MLD meat sample was weighed on an electronic scale sensitive to 0.001 g . The sample was placed in a vacuum-packed plastic bag and cooked at 80 °C for 1 h in a water bath. The meat was then kept at +4 °C for 12 h before being removed from the bag, thoroughly dried with a paper towel, and reweighed. The sample's percentage cooking loss was calculated as the difference in weight before and after cooking (Honikel, 1998).

Meat samples of 50 g were taken from tail fat, liver tissue, and MLD before freezing at -18 °C . Fat samples were extracted from each sample using the Soxhlet Extraction method by AOAC (method no. 960.39). The extracted fats were then stored at -20 °C until analyzed. The fatty acids were converted into fatty acid methyl esters (FAMEs) using 0.5 N methanolic NaOH and 20 % methanolic boron trifloride (BF3). The FAMEs were isolated in n-heptan, determined using a gas chromatograph (Schimadzu GC-2025) and equipped with a Rt-2560 Restek column with 0.25mm×100m×0.20µm dimensions. The column temperature program was started at 100 °C for 2 min before being increased to 250 °C at a rate of 4 ${}^{\circ}C\;\min^{-1}$ for 15 min . The carrier gas was hydrogen, while the gas flow rate was 1.2 $\mathrm{mL}\;\min^{-1}$ , and the split ratio was set at 50:1 . The injection was 1 µL using an automatic sampler. Both the injector and detector temperatures were set at 250 °C . The results for the individual fatty acids in shown the chromatograms are expressed as a percentage of the reference standard (Restek FAME Mix).

### 2.3 Gene expression analysis

For the gene expression analyses, tissue samples of 1 g each were taken from the MLD, liver, and gluteal fat (through the gluteal muscle) and quickly placed in nuclease-free tubes, frozen in liquid nitrogen, and stored at -86 °C . The RNA was then isolated using the Trizol method (Rio et al., 2010) to measure the expression levels of the *SREBP-1c, PPAR* γ , and *FASN* genes in each sample. The purity and concentration levels of the nucleic acids in the isolated RNA samples were evaluated with a Merinton-SMA 1000 device. The RNA quality of each sample was checked via the 28S and 18S bands in 1 % agarose gel electrophoresis (100 V and 25 min ). RNA isolation was repeated for each sample until the appropriate purity, concentration, and non-integrity were achieved. To eliminate genomic DNA contamination before cDNA synthesis, the samples were treated using DNA digestion processes (DNase I, RNase free, Thermo Fisher Scientific, USA, cat. no. 18068015). cDNA synthesis was then conducted using a high-capacity cDNA reverse transcription kit (Thermo Fisher Scientific, USA, Cat No.: 4368814). The post-reaction samples were completed to 200 µL in nuclease-free water and stored at -20 °C until the analyses.

Amplification of the *SREBP-1c*, *PPAR* γ , and *FASN* genes in the liver, MLD, and gluteal fat tissue samples was performed using real-time PCR (Rotor Gene Q, Qiagen). For gene amplification, a kit was used containing SYBR Green I paint (Power SYBR green PCR Master, Thermo Fisher Scientific, USA, cat no.: 4368702). Each example was duplicated. The RT-qPCR reaction comprised 40 cycles of 15 s at 95 °C , 60 s at 60 °C , and 30 s at 72 °C . The ACTB and PPIA genes were used as housekeeping genes for the liver and MLD muscle tissue samples, respectively. Table 2 presents the sequences of the target gene and housekeeping genes. Product control after the real-time PCR application was performed using a 50 bp DNA ladder (Thermo Fisher Scientific, cat. no.: SM0371) in 1.5 % agarose gel electrophoresis (100 V and 45 dk).

**Table 2:** Forward (F) and reverse (R) sequence of primers^∗^.

Gene	F and R sequence of primers	Base
		length
*ACTB*	F: 5'-TTGGATCGAGCATTCCCAAA-3'	165
	R: 5'-ACTGGCCCCTTCTCCTTAGA-3'	
*PPIA*	F: 5'-GCAAAGTGAAAGAGGGCATGAA-3'	87
	R: 5'-CAATGGTGATCTTCTTGCTGGT-3'	
*RPLP0*	F: 5'-TCCTTCTTCCAGGCTTTAGGC-3'	78
	R: 5'-AATCAGCTGCACATCGCTCA-3'	
*SREBP-1c*	F: 5'-GCTACCGCTCTTCCATCAATG-3'	109
	R: 5'-ATGTAGTCGATGGCTTTGCG-3'	
*PPAR* γ	F: 5'-TTGCTGTGGGGATGTCTCAT-3'	215
	R: 5'-TGTCGTCTTTCCCGTCAAGA-3'	
*FASN*	F: 5'-CTTAACAGCACGTCCCCCAT-3'	149
	R: 5'-TCCTCGGGCTTGTCTTGTTC-3'	

^∗^ The primers were designed by authors.

### 2.4 Statistical analysis

Statistical analyses were performed using the SPSS 23.0 package program. Descriptive statistics for each variable were calculated and presented as mean ± standard error of mean (SEM). Before the significance tests, the variables were evaluated in terms of normality and homogeneity of variance using the Shapiro–Wilk and Levene's tests, respectively. The significance of the differences in meat quality characteristics among the three slaughter weight groups was determined using the one-way ANOVA and Tukey multiple comparison tests. The Bonferroni correction was applied after ANOVA to adjust p values for multiple comparisons. While the geometric means were determined for the housekeeping genes, the expression levels of the studied genes were calculated with the 2- ΔΔ Ct method and presented as fold change (Livak and Schmittgen, 2001) and compared to the Gr35 as a control group. A probability value of less than 0.05 was considered to be significant in all of the analyses.

## 3 Results

### 3.1 Meat quality characteristics

Table 3 presents the results of the meat quality analysis. For the MLD muscle, the pH values in the three slaughter weight groups (Gr35, Gr40, and Gr45) were significantly different 15 min after slaughter ( 6.44±0.01 , 6.41±0.01 , and 6.39±0.01 , respectively) ( P<0.001 ) and 24 h after slaughter ( 5.38±0.04 , 5.50±0.03 , and 5.45±02.0 , respectively) ( P=0.049 ). In terms of color, 24 h after slaughtering, the $L^{*}$ and $b^{*}$ values for both the MLD and MSM samples were similar across all three groups, while the $a^{*}$ values showed significant differences in both the MLD ( P=0.006 ) and MSM ( P<0.001 ) groups. Neither water-holding capacity (11.53 %–14.27 %) nor cooking loss (26.22 %–29.25 %) varied significantly between groups.

**Table 3:** Meat quality traits in experimental groups ( X±Sx ). Bold font indicates statistical significance.

Traits		Time after slaughter	Groups			P
			Gr35 ( n=6 )	Gr40 ( n=6 )	Gr45 ( n=6 )	
pH (MLD)		15 min	$6.44\pm 0.01^{a}$	$6.41\pm 0.01^{b}$	$6.39\pm 0.01^{b}$	**0.001**
		1 h	6.24±0.01	6.22±0.01	6.21±0.01	0.068
		24 h	$5.38\pm 0.04^{a}$	$5.50\pm 0.03^{b}$	$5.45\pm 0.02^{\text{ab}}$	**0.049**
		Difference (15 min - 1 h)	0.20±0.02	0.19±0.01	0.18±0.01	0.605
		Difference (15 min - 24 h)	$1.06\pm 0.05^{a}$	$0.91\pm 0.03^{b}$	$0.94\pm 0.02^{\text{ab}}$	**0.019**
pH (MSM)		15 min	6.58±0.07	6.52±0.03	6.49±0.03	0.379
		1 h	6.36±0.03	6.39±0.02	6.30±0.03	0.075
		24 h	5.42±0.02	5.51±0.04	5.52±0.03	**0.046**
		Difference (15 min - 1 h)	0.22±0.05	0.13±0.02	0.18±0.03	0.183
		Difference (15 min - 24 h)	$1.16\pm 0.08^{a}$	$1.01\pm 0.03^{\text{ab}}$	$0.97\pm 0.04^{b}$	**0.045**
Color (MLD)	$L^{*}$	15 min	36.17±0.29	36.69±0.17	37.27±0.24	0.103
		1 h	35.99±0.28	36.24±0.26	36.76±0.47	0.203
		24 h	40.55±0.57	40.60±0.79	41.17±0.23	0.701
	$a^{*}$	15 min	13.46±0.29	13.82±0.36	13.87±0.26	0.321
		1 h	13.85±0.30	13.28±0.39	13.49±0.32	0.490
		24 h	$16.73\pm 0.24^{a}$	$17.04\pm 0.40^{a}$	$18.47\pm 0.37^{b}$	**0.006**
	$b^{*}$	15 min	4.68±0.19	4.32±0.07	4.24±0.15	0.121
		1 h	4.71±0.19	4.19±0.26	4.32±0.16	0.221
		24 h	8.98±0.78	8.80±0.73	8.67±0.40	0.347
Color (MSM)	$L^{*}$	15 min	37.66±0.35	36.68±0.19	37.62±0.42	0.276
		1 h	37.81±0.39	36.44±0.19	36.90±0.35	0.223
		24 h	41.44±0.63	41.94±0.40	42.78±0.30	0.136
	$a^{*}$	15 min	13.92±0.34	14.14±0.49	14.30±0.39	0.092
		1 h	13.75±0.42	14.95±0.59	14.53±0.29	0.194
		24 h	$15.65\pm 0.27^{a}$	$17.95\pm 0.27^{b}$	$18.94\pm 0.37^{b}$	<0.001
	$b^{*}$	15 min	4.69±0.12	4.58±0.13	4.48±0.19	0.137
		1 h	4.85±0.21	4.80±0.20	4.31±0.18	0.249
		24 h	10.02±0.67	9.92±0.41	9.35±0.36	0.602
Water-holding capacity (%)		24 h	11.53±0.45	12.50±0.43	12.93±0.54	0.269
		48 h	13.47±0.68	13.77±0.74	13.67±0.63	0.609
		72 h	14.27±0.91	13.80±1.15	14.10±1.20	0.439
Cooking loss (%)		24 h	27.72±1.14	28.55±0.74	26.48±1.05	0.357
		48 h	28.87±0.80	29.25±1.03	26.22±1.09	0.090

^a,b^ Means with different letters in rows differ significantly ( P<0.05 ).

Table 4 presents the mean values for fatty acid compositions for the different tissues across the three groups. Fatty acid composition varied significantly by tissue type (liver, MLD, and tail fat) ( P<0.001 , P<0.01 , and P<0.05 , respectively), with the exception of C18:0 , C18:3ω3 , C23:0 , and C24:0 . Slaughtering weight had a significant effect on the ratio of C14:0 ( P<0.05 ), C16:0 ( P<0.001 ), C18:2ω6 ( P<0.01 ), C20:1 ( P<0.01 ), and C22:6ω3 ( P<0.05 ) but not on the other fatty acids. The ratio of total unsaturated fatty acids varied significantly for all three tissue types across the three slaughter weight groups in liver at 51.91%±0.64 %, 52.34%±1.46 %, and 53.12%±1.47 %, respectively; in MLD at 50.34%±1.31 %, 49.08%±1.29 %, and 45.61%±1.30 %, respectively; and in tail fat at 47.73%±1.73 %, 54.67%±1.57 %, and 50.42%±4.29 %, respectively. Table 5 presents the sum and index values for the meat quality characteristics across the three groups. While the effect of tissue type was found to be significant for all characteristics at various levels of statistical significance ( P<0.05 , P<0.01 , and P<0.001 ), the group × tissue interaction showed significant differences for only the NV and TI values ( P=0.001 and P=0.035 , respectively).

**Table 4:** Means of fatty acid compositions in experimental groups and different tissues (%) ( X±Sx ). Bold font indicates statistical significance.

Fatty acids	Groups	Tissues				P		
		Liver	MLD	Tail fat	General means	Groups ( G )	Tissues ( T )	G×T
C10:0	Gr35	0.07±0.01	0.30±0.01	0.53±0.09	0.30±0.05	0.425	<0.001	0.841
	Gr40	0.06±0.01	0.25±0.02	0.45±0.07	0.25±0.04			
	Gr45	0.08±0.02	0.28±0.05	0.43±0.05	0.26±0.04			
	Gen. means	$0.07\pm 0.01^{c}$	$0.28\pm 0.02^{b}$	$0.47\pm 0.04^{a}$				
C12:0	Gr35	0.06±0.01	0.26±0.04	0.33±0.04	0.22±0.03	0.308	<0.001	0.450
	Gr40	0.06±0.01	0.19±0.02	0.30±0.06	0.18±0.03			
	Gr45	0.09±0.03	0.20±0.08	0.23±0.02	0.17±0.03			
	Gen. means	$0.07\pm 0.01^{b}$	$0.22\pm 0.03^{a}$	$0.29\pm 0.03^{a}$				
C13:0	Gr35	0.20±0.01	0.03±0.01	0.12±0.04	0.11±0.02	0.064	<0.001	0.627
	Gr40	0.17±0.02	0.01±0.001	0.07±0.01	0.09±0.02			
	Gr45	0.21±0.02	0.01±0.003	0.11±0.02	0.11±0.02			
	Gen. means	$0.19\pm 0.01^{a}$	$0.02\pm 0.003^{c}$	$0.10\pm 0.02^{b}$				
C14:0	Gr35	1.96±0.15	4.82±0.49	2.15±0.44	$2.97\pm 0.38^{A}$	**0.021**	**0.001**	0.084
	Gr40	1.64±0.06	2.14±0.55	1.79±0.48	$1.85\pm 0.22^{B}$			
	Gr45	2.04±0.21	2.88±1.06	1.87±0.57	$2.26\pm 0.40^{\mathrm{AB}}$			
	Gen. means	$1.86\pm 0.09^{b}$	$3.30\pm 0.48^{a}$	$1.94\pm 0.27^{b}$				
C14:1	Gr35	1.09±0.07	0.10±0.01	1.41±0.11	0.87±0.14	0.713	<0.001	0.572
	Gr40	1.01±0.04	0.17±0.03	1.23±0.20	0.81±0.12			
	Gr45	1.13±0.09	0.14±0.02	1.16±0.20	0.81±0.14			
	Gen. means	$1.07\pm 0.04^{a}$	$0.14\pm 0.01^{b}$	$1.27\pm 0.10^{a}$				
C15:0	Gr35	0.21±0.01	0.11±0.05	1.77±0.25	0.69±0.20	0.905	<0.001	0.905
	Gr40	0.24±0.03	0.15±0.06	1.57±0.16	0.63±0.16			
	Gr45	0.21±0.02	0.17±0.10	1.53±0.42	0.64±0.22			
	Gen. means	$0.22\pm 0.01^{b}$	$0.14\pm 0.04^{b}$	$1.63\pm 0.15^{a}$				
C15:1	Gr35	0.39±0.03	0.10±0.03	1.23±0.10	0.58±0.12	0.125	<0.001	0.110
	Gr40	0.34±0.03	0.12±0.01	0.90±0.14	0.45±0.09			
	Gr45	0.42±0.03	0.07±0.02	1.46±0.33	0.65±0.19			
	Gen. means	$0.38\pm 0.02^{b}$	$0.10\pm 0.01^{c}$	$1.18\pm 0.12^{a}$				
C16:0	Gr35	$20.92\pm 0.6^{b}$	$26.13\pm 0.58^{\mathrm{aAB}}$	$22.76\pm 1.58^{\mathrm{abA}}$	23.27±0.77	<0.001	<0.001	**0.038**
	Gr40	$20.76\pm 0.70^{b}$	$24.79\pm 0.57^{\mathrm{aB}}$	$17.42\pm 1.52^{\mathrm{bB}}$	20.98±0.88			
	Gr45	$21.34\pm 0.42^{b}$	$29.61\pm 1.52^{\mathrm{aA}}$	$24.89\pm 2.00^{\mathrm{bA}}$	25.28±1.20			
	Gen. means	20.97±0.36	26.68±0.70	21.50±1.20				
C16:1	Gr35	4.01±0.16	3.35±0.11	13.53±2.47	6.96±1.37	0.744	<0.001	0.822
	Gr40	3.81±0.10	3.25±0.30	16.49±3.08	7.63±1.69			
	Gr45	4.27±0.21	3.68±0.62	14.52±2.51	7.49±1.55			
	Gen. means	$4.00\pm 0.09^{b}$	$3.41\pm 0.20^{b}$	$14.86\pm 1.51^{a}$				
C17:0	Gr35	2.18±0.14	0.80±0.04	2.08±0.21	1.68±0.17	0.473	<0.001	0.227
	Gr40	2.20±0.17	1.15±0.23	1.73±0.28	1.72±0.16			
	Gr45	2.11±0.16	1.07±0.25	2.46±0.33	1.88±0.21			
	Gen. means	$2.16\pm 0.09^{a}$	$1.00\pm 0.11^{b}$	$2.07\pm 0.16^{a}$				
C17:1	Gr35	1.02±0.07	0.66±0.05	1.84±0.15	1.18±0.13	0.900	<0.001	0.638
	Gr40	0.94±0.03	0.93±0.24	1.72±0.18	1.18±0.12			
	Gr45	1.07±0.08	0.94±0.09	1.70±0.35	1.24±0.15			
	Gen. means	$1.00\pm 0.03^{b}$	$0.84\pm 0.09^{b}$	$1.76\pm 0.13^{a}$				
C18:0	Gr35	21.46±0.48	17.11±1.45	22.36±0.78	20.31±0.77	0.110	0.607	0.147
	Gr40	21.67±0.88	22.15±1.44	21.90±1.84	21.89±0.76			
	Gr45	19.74±1.08	20.12±1.48	17.96±3.20	19.28±1.16			
	Gen. means	21.06±0.49	19.78±0.95	20.90±1.19				

**Table 4:** Continued.

Fatty acids	Groups	Tissues				P		
		Liver	MLD	Tail fat	General means	Groups ( G )	Tissues ( T )	G×T
C18:1	Gr35	$28.32\pm 0.64^{a}$	$36.10\pm 1.19^{b}$	$24.96\pm 2.56^{\mathrm{aA}}$	29.79±1.45	0.205	**0.014**	**0.003**
	Gr40	$28.15\pm 0.56^{a}$	$32.15\pm 1.61^{\mathrm{ab}}$	$34.68\pm 2.22^{\mathrm{bB}}$	31.47±1.06			
	Gr45	29.12±1.29	30.06±1.23	$28.12\pm 3.49^{A}$	29.10±1.23			
	Gen. means	28.47±0.45	32.93±0.97	29.32±1.80				
C18:2 ω6	Gr35	9.47±0.50	2.86±0.15	2.42±0.23	$4.92\pm 0.80^{A}$	**0.002**	<0.001	0.523
	Gr40	9.18±0.25	2.60±0.23	1.97±0.29	$4.83\pm 0.80^{A}$			
	Gr45	8.30±0.19	1.87±0.16	1.97±0.10	$4.05\pm 0.81^{B}$			
	Gen. means	$9.03\pm 0.22^{a}$	$2.47\pm 0.14^{b}$	$2.13\pm 0.14^{b}$				
C20:0	Gr35	0.14±0.02	0.05±0.01	0.15±0.06	0.11±0.02	0.296	<0.001	0.275
	Gr40	0.17±0.01	0.03±0.01	0.08±0.03	0.10±0.02			
	Gr45	0.15±0.02	0.03±0.01	0.07±0.01	0.08±0.01			
	Gen. means	$0.15\pm 0.01^{a}$	$0.04\pm 0.005^{b}$	$0.10\pm 0.02^{c}$				
C18:3 ω6	Gr35	0.58±0.03	0.33±0.04	0.33±0.09	0.41±0.04	0.114	<0.001	0.708
	Gr40	0.56±0.01	0.28±0.02	0.39±0.04	0.42±0.03			
	Gr45	0.47±0.01	0.24±0.04	0.31±0.07	0.34±0.04			
	Gen. means	$0.54\pm 0.02^{a}$	$0.29\pm 0.02^{b}$	$0.35\pm 0.04^{b}$				
C20:1	Gr35	0.38±0.05	0.22±0.03	0.29±0.06	$0.30\pm 0.03^{A}$	**0.004**	<0.001	0.309
	Gr40	0.28±0.02	0.14±0.02	0.17±0.02	$0.20\pm 0.02^{B}$			
	Gr45	0.32±0.03	0.12±0.04	0.31±0.03	$0.25\pm 0.03^{\mathrm{AB}}$			
	Gen. means	$0.33\pm 0.02^{a}$	$0.16\pm 0.02^{b}$	$0.25\pm 0.03^{a}$				
C18:3 ω3	Gr35	0.29±0.02	0.17±0.03	0.33±0.07	0.26±0.03	0.640	0.069	0.759
	Gr40	0.25±0.01	0.20±0.03	0.23±0.07	0.23±0.02			
	Gr45	0.27±0.03	0.17±0.04	0.24±0.12	0.22±0.04			
	Gen. means	0.27±0.01	0.18±0.02	0.27±0.05				
C20:2 ω6	Gr35	0.14±0.01	0.01±0.004	0.01±0.001	0.05±0.02	0.895	<0.001	0.941
	Gr40	0.14±0.01	0.01±0.003	0.01±0.002	0.06±0.02			
	Gr45	0.15±0.02	0.01±0.002	0.01±0.003	0.06±0.02			
	Gen. means	$0.15\pm 0.01^{a}$	$0.01\pm 0.002^{b}$	$0.01\pm 0.001^{b}$				
C22:0	Gr35	0.76±0.02	0.03±0.002	0.02±0.003	0.27±0.08	0.554	<0.001	0.766
	Gr40	0.68±0.08	0.02±0.002	0.02±0.003	0.28±0.08			
	Gr45	0.71±0.05	0.02±0.01	0.01±0.004	0.25±0.09			
	Gen. means	$0.72\pm 0.03^{a}$	$0.02\pm 0.002^{b}$	$0.02\pm 0.002^{b}$				
C20:3 ω6	Gr35	0.60±0.04	0.09±0.01	0.02±0.005	0.24±0.06	0.777	<0.001	0.911
	Gr40	0.59±0.05	0.06±0.01	0.02±0.01	0.25±0.07			
	Gr45	0.60±0.05	0.04±0.01	0.02±0.01	0.22±0.07			
	Gen. means	$0.60\pm 0.03^{a}$	$0.07\pm 0.01^{b}$	$0.02\pm 0.003^{b}$				
C22:1	Gr35	0.05±0.01	0.01±0.003	0.02±0.002	0.03±0.01	0.453	<0.001	0.885
	Gr40	0.04±0.005	0.01±0.002	0.02±0.01	0.02±0.004			
	Gr45	0.04±0.005	0.01±0.003	0.01±0.004	0.02±0.01			
	Gen. means	$0.04\pm 0.004^{a}$	$0.01\pm 0.002^{b}$	$0.01\pm 0.002^{b}$				
C20:3 ω3	Gr35	0.08±0.01	0.05±0.004	0.01±0.002	0.05±0.01	0.150	<0.001	0.304
	Gr40	0.07±0.01	0.03±0.01	0.02±0.004	0.04±0.01			
	Gr45	0.06±0.004	0.02±0.01	0.02±0.01	0.04±0.01			
	Gen. means	$0.07\pm 0.01^{a}$	$0.03\pm 0.004^{b}$	$0.01\pm 0.003^{c}$				
C20:4 ω6	Gr35	5.57±0.17	0.53±0.04	0.05±0.01	1.83±0.66	0.795	<0.001	0.992
	Gr40	5.42±0.57	0.31±0.05	0.07±0.01	2.50±0.71			
	Gr45	5.36±0.38	0.19±0.04	0.03±0.01	2.14±0.75			
	Gen. means	$5.44\pm 0.27^{a}$	$0.35\pm 0.04^{b}$	$0.05\pm 0.01^{b}$				

**Table 4:** Continued.

Fatty acids	Groups	Tissues				P		
		Liver	MLD	Tail fat	General means	Groups ( G )	Tissues ( T )	G×T
C23:0	Gr35	0.11±0.09	0.002±0.0007	0.001±0.0001	0.04±0.03	0.547	0.068	0.639
	Gr40	0.01±0.001	0.002±0.0004	0.002±0.0005	0.004±0.001			
	Gr45	0.09±0.08	0.002±0.0003	0.002±0.0005	0.03±0.03			
	Gen. means	0.06±0.04	0.002±0.0003	0.002±0.0002				
C22:2 ω6	Gr35	0.46±0.09	0.02±0.01	0.002±0.0004	0.16±0.06	0.347	<0.001	0.350
	Gr40	0.44±0.03	0.004±0.001	0.001±0.0005	0.16±0.05			
	Gr45	0.32±0.08	0.01±0.01	0.002±0.0003	0.11±0.05			
	Gen. means	$0.41\pm 0.04^{a}$	$0.01\pm 0.005^{b}$	$0.002\pm 0.0002^{b}$				
C24:0	Gr35	0.02±0.01	0.03±0.01	0.003±0.0006	0.02±0.004	0.423	0.207	0.234
	Gr40	0.01±0.001	0.02±0.004	0.01±0.002	0.01±0.002			
	Gr45	0.12±0.11	0.01±0.004	0.002±0.001	0.04±0.04			
	Gen. means	0.04±0.03	0.02±0.004	0.004±0.0008				
C20:5 ω3	Gr35	$0.03\pm 0.02^{B}$	0.003±0.0008	0.009±0.001	0.01±0.01	0.068	**0.001**	**0.029**
	Gr40	$0.07\pm 0.06^{B}$	0.007±0.003	0.004±0.001	0.03±0.02			
	Gr45	$0.25\pm 0.10^{\mathrm{aA}}$	$0.004\pm 0.002^{b}$	$0.004\pm 0.001^{b}$	0.09±0.04			
	Gen. means	0.11±0.04	0.004±0.001	0.006±0.0008				
C24:1	Gr35	0.39±0.04	0.03±0.003	0.005±0.001	0.14±0.05	0.530	<0.001	0.509
	Gr40	0.31±0.06	0.02±0.003	0.007±0.001	0.12±0.04			
	Gr45	0.27±0.09	0.03±0.02	0.02±0.01	0.11±0.04			
	Gen. means	$0.33\pm 0.04^{a}$	$0.03\pm 0.01^{b}$	$0.009\pm 0.003^{b}$				
C22:6 ω3	Gr35	$0.89\pm 0.02^{\mathrm{aA}}$	$0.16\pm 0.01^{b}$	$0.11\pm 0.002^{b}$	0.39±0.09	**0.028**	<0.001	**0.025**
	Gr40	$0.74\pm 0.07^{\mathrm{aB}}$	$0.14\pm 0.003^{b}$	$0.12\pm 0.003^{b}$	0.35±0.07			
	Gr45	$0.69\pm 0.04^{\mathrm{aB}}$	$0.12\pm 0.004^{b}$	$0.14\pm 0.03^{b}$	0.31±0.07			
	Gen. means	0.77±0.04	0.14±0.005	0.12±0.01				

^a,b,c^ Means with different letters in rows differ significantly ( P<0.05 ).^A,B^ Means with different letters in columns differ significantly ( P<0.05 ).

**Table 5:** Sums and ratios based on fatty acid in experimental groups and different tissues (%) ( X±Sx ). Bold font indicates statistical significance.

Traits	Groups	Tissues				P		
		Liver	MLD	Tail fat	General means	Groups ( G )	Tissues ( T )	G×T
Σ SFA	Gr35	48.09±0.64	49.66±1.31	52.27±1.73	50.01±0.82	0.233	**0.027**	0.092
	Gr40	47.66±1.46	50.92±1.29	45.33±1.57	47.95±0.95			
	Gr45	46.88±1.47	54.39±1.30	49.58±4.29	50.29±1.68			
	Gen. means	$47.59\pm 0.70^{b}$	$51.49\pm 0.86^{a}$	$49.03\pm 1.58^{\mathrm{ab}}$				
Σ MUFA	Gr35	35.65±0.84	46.12±1.34	44.46±1.60	42.08±1.32	0.315	<0.001	0.065
	Gr40	34.88±0.68	45.45±1.13	51.87±1.73	43.58±1.80			
	Gr45	36.64±1.42	42.93±1.18	47.69±4.38	42.42±1.90			
	Gen. means	$35.62\pm 0.54^{b}$	$44.95\pm 0.74^{a}$	$48.03\pm 1.62^{a}$				
Σ PUFA	Gr35	16.26±0.89	4.22±0.19	3.27±0.30	7.92±1.46	0.298	<0.001	0.349
	Gr40	17.47±0.93	3.63±0.28	2.79±0.39	8.46±1.66			
	Gr45	16.48±0.51	2.68±0.24	2.72±0.15	7.29±1.75			
	Gen. means	$16.79\pm 0.48^{a}$	$3.56\pm 0.20^{b}$	$2.94\pm 0.18^{b}$				
Σ UFA	Gr35	51.91±0.64	50.34±1.31	47.73±1.73	49.99±0.82	0.233	**0.027**	0.092
	Gr40	52.34±1.46	49.08±1.29	54.67±1.57	52.05±0.95			
	Gr45	53.12±1.47	45.61±1.30	50.42±4.29	49.71±1.68			
	Gen. means	$52.41\pm 0.70^{a}$	$48.50\pm 0.86^{b}$	$50.97\pm 1.58^{\mathrm{ab}}$				
ΣPUFA/ΣSFA	Gr35	0.34±0.02	0.09±0.004	0.06±0.01	0.16±0.03	0.514	<0.001	0.496
	Gr40	0.37±0.03	0.07±0.01	0.06±0.01	0.18±0.04			
	Gr45	0.35±0.02	0.05±0.01	0.06±0.004	0.15±0.04			
	Gen. means	$0.36\pm 0.01^{a}$	$0.07\pm 0.005^{b}$	$0.06\pm 0.004^{b}$				
ΣUFA/ΣSFA	Gr35	1.08±0.03	1.02±0.05	0.92±0.06	1.01±0.03	0.354	**0.048**	0.160
	Gr40	1.11±0.06	0.97±0.05	1.22±0.08	1.10±0.04			
	Gr45	1.14±0.07	0.84±0.04	1.09±0.22	1.03±0.08			
	Gen. means	$1.11\pm 0.03^{a}$	$0.95\pm 0.03^{b}$	$1.08\pm 0.08^{\mathrm{ab}}$				
ω6	Gr35	14.97±0.88	3.84±0.16	2.81±0.32	7.21±1.37	0.251	<0.001	0.258
	Gr40	16.34±0.85	3.26±0.29	2.43±0.34	7.82±1.57			
	Gr45	15.20±0.48	2.36±0.23	2.34±0.12	6.63±1.63			
	Gen. means	$15.57\pm 0.46^{a}$	$3.20\pm 0.20^{b}$	$2.54\pm 0.17^{b}$				
ω3	Gr35	1.29±0.02	0.38±0.04	0.46±0.07	0.71±0.10	0.379	<0.001	0.751
	Gr40	1.13±0.12	0.37±0.03	0.36±0.08	0.65±0.10			
	Gr45	1.28±0.12	0.32±0.05	0.39±0.12	0.66±0.13			
	Gen. means	$1.22\pm 0.06^{a}$	$0.36\pm 0.02^{b}$	$0.40\pm 0.05^{b}$				
ω6/ω3	Gr35	11.62±0.69	10.55±0.76	7.41±1.94	9.86±0.81	0.445	<0.001	0.223
	Gr40	15.27±1.24	9.17±1.40	7.44±0.83	10.87±1.04			
	Gr45	12.36±1.21	7.87±0.88	7.76±1.49	9.33±0.87			
	Gen. means	$13.24\pm 0.71^{a}$	$9.27\pm 0.64^{b}$	$7.52\pm 0.81^{b}$				
NV	Gr35	2.40±0.09	2.04±0.05	$2.15\pm 0.23^{B}$	2.20±0.09	<0.001	**0.003**	**0.001**
	Gr40	$2.41\pm 0.07^{b}$	$2.20\pm 0.06^{b}$	$3.40\pm 0.36^{\mathrm{aA}}$	2.66±0.16			
	Gr45	2.29±0.07	1.72±0.11	$1.92\pm 0.24^{B}$	1.98±0.11			
	Gen. means	2.37±0.05	2.00±0.06	2.52±0.22				
AI	Gr35	0.57±0.02	0.73±0.05	0.66±0.05	0.65±0.03	0.166	**0.008**	0.818
	Gr40	0.55±0.03	0.63±0.05	0.54±0.07	0.57±0.03			
	Gr45	0.53±0.04	0.70±0.06	0.54±0.10	0.59±0.04			
	Gen. means	$0.55\pm 0.02^{b}$	$0.69\pm 0.03^{a}$	$0.58\pm 0.04^{b}$				
TI	Gr35	1.02±0.02	1.09±0.06	$1.29\pm 0.11^{A}$	1.13±0.05	0.198	**0.024**	**0.035**
	Gr40	1.03±0.06	1.19±0.07	$0.91\pm 0.06^{B}$	1.04±0.04			
	Gr45	$0.98\pm 0.06^{b}$	$1.37\pm 0.09^{a}$	$1.18\pm 0.19^{\mathrm{abAB}}$	1.17±0.08			
	Gen. means	1.01±0.03	1.21±0.05	1.12±0.08				

^a,b^ Means with different letters in rows differ significantly ( P<0.05 ).^A,B^ Means with different letters in columns differ significantly ( P<0.05 ).NV =(C18:0+C18:1)/C16:0 .AI =(C12:0+(4⋅C14:0)+C18:0)/Σ UFA.TI =(C14:0+C16:0+C18:0)/(0.5⋅C18:1)+(0.5⋅ΣPUFA)+(0.5⋅Σω6)+(3⋅Σω3)+(Σω3/Σω6) .

### 3.2 Gene expression

The RNA isolates from liver ( A260/280=1.81±0.01 ; concentration 713.18±19.55 $\mathrm{ng}\;µL^{-1}$ ), MLD muscle ( A260/280=1.85±0.01 ; concentration 555.88±33.29 $\mathrm{ng}\;µL^{-1}$ ), and gluteal fat ( A260/280=1.74±0.01 ; concentration 555.46±37.32 $\mathrm{ng}\;µL^{-1}$ ) were sufficiently pure and concentrated for cDNA synthesis and gene expression analysis.

Table 6 presents the expression values of the target genes in liver and MLD muscle tissue for each slaughter weight group (Gr35, Gr40, and Gr45). In liver tissue, *SREBP-1c* and *PPAR* γ gene expression did not differ significantly across groups. For *FASN*, no statistically significant increase in gene expression was observed in any of the three tissues (liver, MLD, and gluteal fat) in the Gr40 group; however, for Gr45, *FASN* expression in the liver was statistically significantly downregulated compared to the Gr35 group ( P=0.04 ). In MLD muscle tissue, while *SREBP-1c* and *PPAR* γ gene expression did not show a statistically significant difference between groups, a statistically significant difference was observed in *FASN* expression ( P<0.02 ).

**Table 6:** Target gene expression of experimental groups ( X±Sx ). Bold font indicates statistical significance.

Tissues	Gene	Groups		
		Gr35	Gr40	Gr45
Liver	*SREBP-1c*	1.00±0.23	1.54±0.18	0.95±0.12
			P=0.11	P=0.87
	*PPAR* γ	1.00±0.26	1.31±0.21	1.26±0.25
			P=0.38	P=0.48
	*FASN*	1.00±0.25	1.03±0.24	0.36±0.12
			P=0.92	P=0.04
MLD	*SREBP-1c*	1.00±0.16	1.52±0.39	1.25±0.64
			P=0.20	P=0.64
	*PPAR* γ	1.00±0.13	0.72±0.25	1.20±0.35
			P=0.31	P=0.60
	*FASN*	1.00±0.26	1.89±0.61	2.01±0.24
			P=0.18	P=0.02
Gluteal fat	*SREBP-1c*	1.00±0.10	1.33±0.27	0.84±0.29
			P=0.24	P=0.62
	*PPAR* γ	1.00±0.27	0.63±0.10	0.72±0.12
			P=0.23	P=0.37
	*FASN*	1.00±0.19	1.64±0.14	0.72±0.28
			P=0.15	P=0.43

## 4 Discussion

### 4.1 Meat quality characteristics

One of the most important factors affecting the taste, tenderness, and juiciness of meat is pH, which should ideally range between 5.50 and 5.80 in the 24 h after slaughtering to yield high-quality meat (Mercan et al., 2022). Meat pH values decrease after slaughter, given appropriate storage conditions, which ensures that the meat becomes more tender and juicy, thereby improving its quality.

For the Lalahan genotype lambs used in the present study, the MLD pH values in the Gr35, Gr40, and Gr45 groups after 15 min (6.44, 6.41, and 6.39, respectively), 1 h (6.24, 6.22, and 6.21, respectively), and 24 h (5.38, 5.50, and 5.45, respectively) conform to normally accepted values for these measurement times (Ferguson and Warner, 2008; Mercan et al., 2022; Yakan and Ünal, 2010). That is, the pH values measured in the MLD and MSM muscles at different slaughtering weights decreased as the time after slaughtering increased. The low pH values in all three groups 24 h after slaughtering were similar across the three groups for both MLD (1.06, 0.91, and 0.94, respectively) and the MSM (1.16, 1.01, and 0.97, respectively).

In the present study, the fall in pH over time was smaller as the animals' slaughter weight increased. However, previous findings vary regarding the effect of slaughter weight on pH . As in the present study, several studies reported smaller post-slaughter falls in meat pH in lambs of various breeds with higher slaughter weight (Cañeque et al., 2005; Dıaz et al., 2002), whereas other studies of several Spanish breeds and Merino sheep reported the opposite (Martínez-Cerezo et al., 2005; Tejeda et al., 2008).

To test the effect of slaughter weight on meat color in MLD and MSM muscles, three color coordinates ( $L^{*}$ , $a^{*}$ , $b^{*}$ ) were measured at three times after slaughter (15 min and 60 and 24 h ). The only significant effect of slaughter weight on these coordinates was for $a^{*}$ (redness) ( P<0.01 for MLD; P<0.001 for MSM), which increased with slaughter weight, whereas $L^{*}$ (brightness) and $b^{*}$ (yellowness) did not vary significantly. Regarding the effect of time on meat color, $L^{*}$ , $a^{*}$ , and $b^{*}$ all increased with time after slaughter. The increase in redness observed in the present study is consistent with previous reports showing that myoglobin concentration increases with animal age and physiological maturity. In a large commercial study involving more than 8000 lambs, Calnan et al. (2016) demonstrated that muscle characteristics associated with animal maturity, particularly myoglobin concentration, are major determinants of fresh lamb color. Although ultimate pH exerted an important influence on redness, increasing animal maturity was also associated with changes in muscle pigment characteristics that contribute to darker and redder meat. A review by Prache et al. (2022) further emphasized that meat color is affected by several intrinsic factors, including animal age, muscle fiber type, and myoglobin concentration. Light suckling lambs typically possess low myoglobin concentrations and therefore produce paler meat, whereas increasing biological age results in progressive accumulation of myoglobin and enhanced redness, particularly during the early stages of postnatal growth.

Water release from meat after slaughtering is increased by the contractions in muscle cells and mobilization of water in extracellular cavities due to proteolysis. In the present study, slaughtering weight had no significant impact on water-holding capacity after 24, 48, or 72 h , although percentages increased with time at 24 h (11.53 %–12.93 %), 48 h (13.47 %–13.77 %), and 72 h (13.80 %–14.27 %). These findings are similar to those reported by Yakan and Ünal (2010) for Bafra lambs. The water-holding capacity of MLD muscle at 24 h in the present study was similar to that (12.21 %) reported for lambs slaughtered at 42 kg (Ekiz et al., 2009) but higher than the percentages reported for MLD muscle in Akkaraman, Bafra, and Bafra × Akkaraman F1 lambs slaughtered at 34 and 42 kg (Yaranoğlu and Özbeyaz, 2019).

Cooking loss in meat can be influenced by cooking method, temperature, length, and muscle type. In particular, higher temperatures and longer cooking times increase cooking loss due to denaturation of tightening muscle fibers and a reduction in intercellular gaps. Approximately 70 %–80 % of cooking loss is caused by fat removal during cooking (Santos et al., 2007; Bonvillani et al., 2010). In the present study, slaughtering weight had no significant impact on 24 or 48 h cooking losses. There was also no significant difference in cooking loss if the meat was cooked 24 or 48 h after (26.48 %–28.55 % and 26.22 %–28.87 %, respectively). Similar findings were reported for Bafra (Yakan Ünal, 2010b), Akkaraman, Bafra, and Bafra × Akkaraman F1 lambs (Yaranoglu and Özbeyaz, 2019).

Fatty acid composition varies in different tissues of the body depending on factors such as breed or genotype, nutrition, slaughtering weight, and age (Erol and Ünal, 2021; Yakan et al., 2024). The present study examined fatty acid composition in liver, MLD, and tail fat tissues. MLD fatty acid composition has been widely studied. In our study, we also studied MLD composition in liver and tail fat tissue. However, no study has investigated fatty acid profiles in liver in Turkish indigenous sheep breeds, and there is only limited research on tail fat (Ateş et al., 2025; Tüfekci et al., 2021).

In the present study, the most prevalent fatty acid across all three slaughter weight groups and tissues was oleic acid ( C18:1 ), followed by palmitic acid ( C16:0 ) and stearic acid ( C18:0 ). Slaughter weight had a significant effect on the prevalence of C14:0 , C16-0, C18:2ω6 , C20:1 , and C22:6ω3 . In all tissues studied, a higher slaughter weight was associated with higher palmitic acid ( C16:0 ) percentages, particularly in the Gr45 group, but lower C18:2ω6 and C22:6ω3 percentages. Tissues with more than 1 % total fatty acids had the highest levels of C14:0 , C18:0 , and C18:2ω6 in liver tissue, the highest levels of C14:0 , C16:0 , and C18:1 in MLD tissue, and the highest levels of C14:1 , C15C:0 , C15:1 , C16:1 , and C17:1 in tail fat.

Increasing slaughter weight did not significantly affect the ratio of total saturated fatty acids (SFAs), total single unsaturated fatty acids (PUFAs), or total polyunsaturated fatty acids (PUFAs). SFA and unsaturated fatty acid (UFA) ratios were similar across Gr35, Gr40, and Gr45 (1.01, 1.10, and 1.03, respectively). While this is a positive finding in terms of nutrition, the PUFA/SFA ratios in all three slaughter weight groups were lower than 0.4 (0.16, 0.18, and 0.15, respectively), which is the recommended minimal PUFA/SFA ratio (Jimenez-Colmenero et al., 2017), indicating a nutritional disadvantage in these animals. However, previous findings for many native sheep genotypes have also reported PUFA/SFA ratios of 0.1–0.2 (Yakan and Ünal, 2010; Yaranoglu and Özbeyaz, 2019).

SFA, MUFA, and PUFA levels varied significantly by tissue type (liver, tail fat, and MLD). SFAs the lowest levels (47.59 %, 49.03 %, and 51.49 %, respectively), followed by UFAs (52.41 %, 50.97 %, and 48.50 %, respectively). For PUFA/SFA , the liver sample value (0.36) was close to the recommended level (0.4), whereas the MLD and tail fat values were low (0.07 and 0.06, respectively). The UFA / SFA ratios were similar across the three tissue types (1.11, 0.95, and 1.08, respectively).

In human nutrition, fatty acids have been assessed using various criteria, including ω6/ω3 , nutritive value (NV), atherogenic index (AI), and thrombogenic index (TI) (HMSO, 1994). In the present study, the ω6/ω3 ratio exceeded the recommended value in all three slaughter weight groups (9.86, 10.87, and 9.33, respectively). Similarly, the ω6/ω3 ratios exceeded the recommended value in liver, MLD, and tail fat (13.24, 9.27, and 7.52, respectively).

Nutritional value (NV) is calculated from the ratio of the three most commonly found fatty acids ( C18:0+C18:1/C16:0 ). Of these, palmatic acid ( C16:0 ) increases blood cholesterol levels, whereas oleic acid ( C18:1 ) reduces it. Stearic acid ( C18:0 ) is a saturated fat but does not affect cholesterol levels. C18:1 is also obtained from C18:0 during metabolism (Banskalieva et al., 2000). NV and the NV ratio varied significantly across the three slaughter weight groups, being highest in Gr40 (2.66), followed by Gr35 (2.20) and Gr45 (1.98). NV also differed significantly across tissue types, with the highest level in liver samples (2.37), followed by tail fat (2.52) and MLD (2.00).

While AI is used to assess atherosclerosis risk, TI is used for thrombosis risk. Both should be less than 1 (Ulbricht and Southgate, 1991). In the present study, slaughter weight had no significant effect on AI or TI values and so was insignificant. AI values were below 1 in all three groups (0.65, 0.57, and 0.59, respectively), while TI values were close to 1 (1.13, 1.04, and 1.17, respectively), which are positive findings in terms of nutrition. AI and TI ratios varied significantly by tissue type. AI values in liver, MLD, and tail fat were below 1 (0.55, 0.69, and 0.58, respectively), while TI values were close to 1 (1.01, 1.21, and 1.12, respectively).

### 4.2 Gene expression

Energy metabolism is controlled by complex molecular mechanisms that involve a large number of genes. The primary organ for anabolic and catabolic metabolism is the liver (Deng et al., 2018). *SREBP-1c* is a key transcription factor in carbohydrate and lipid metabolism (Fernández-Alvarez et al., 2011), specifically in de novo lipogenesis in liver tissue. In the present study, *SREBP-1c* mRNA levels in the liver samples did not vary significantly across groups, indicating that its activity was similar regardless of slaughter weight.

As with *SREBP-1c*, the gene expression level of *PPAR* γ , another gene studied for its lipogenesis-associated activity, did not change significantly across slaughtering weights. In ruminants, *PPAR* γ activity is important for glucose and lipid absorption and transportation (Brooks et al., 2015; Lehrke and Lazar, 2005), while *PPAR* γ regulates the activity of many genes associated with lipogenesis, fatty acid absorption, transportation, extension, and hydrolysis (Deng et al., 2018). The similar levels of these transcription factors across the three weight groups may be due to the balance factor in lipogenesis–lipolysis regulation in the liver tissue at different slaughter weights (Yakan et al., 2018).

The *FASN* is responsible for the synthesis of short-, medium-, and long-chain fatty acids, and this gene, which affects saturated fatty acid levels, plays a role in the formation of meat quality (Izadi et al., 2016; Yakan et al., 2021). In the present study, *FASN* expression was higher for Gr40 tissues than for Gr35 and was upregulated 2-fold in Gr45 animals. Deng et al. (2018) reported that C20:5ω3 fatty acid levels in the liver increase as the slaughter weight increases. Upregulation of *FASN*, which is closely associated with fatty acid metabolism, indicates that there are significant changes in fatty acid metabolism in the liver with slaughter weight. Although the animals experienced similar environmental conditions, particularly diet, these differences across slaughter weight groups in gene expression and fatty acid profiles may be associated with differences in weight gain and growth.

*SREBP-1c* and *PPAR* γ gene expression levels in muscle and liver tissue did not differ significantly across weight groups. However, *FASN* gene expression levels in liver were downregulated approximately 3-fold in the Gr45 group. It is known that anabolic-active genes vary significantly depending on growth factors in the body. Reduced *FASN* gene expression in muscle tissue has been associated with higher growth, and so reduced expression of this gene may be linked to slower growth in the Gr45 animals. Furthermore, the relationship between *FASN* gene activity and fatty acid composition is important (Izadi et al., 2016). Our findings showed that, while muscle tissue fatty acid profile did not vary significantly across groups, there were significant differences in the ratio of saturated and unsaturated fatty acids.

## 5 Conclusion

The present study provides new information regarding the effects of slaughter weight on meat quality characteristics, fatty acid composition, and the expression of lipid-metabolism-related genes in Lalahan lambs. The results indicate that slaughter weight had limited effects on most meat quality traits but significantly influenced selected fatty acids and *FASN* expression. These findings contribute to a better understanding of lipid metabolism during growth in sheep and may support optimization of slaughter strategies in intensive lamb production systems. Nevertheless, direct comparisons with other breeds should be confirmed through studies conducted under identical experimental conditions. Given the conditions of the Central Anatolian Region, the Lalahan breed offers potential for high-quality meat production. A slaughter weight of 40 kg can be recommended as appropriate.

## Data Availability

All data can be obtained from the corresponding author upon reasonable request.
